# Climate Change and Future Pollen Allergy in Europe

**DOI:** 10.1289/EHP173

**Published:** 2016-08-24

**Authors:** Iain R. Lake, Natalia R. Jones, Maureen Agnew, Clare M. Goodess, Filippo Giorgi, Lynda Hamaoui-Laguel, Mikhail A. Semenov, Fabien Solomon, Jonathan Storkey, Robert Vautard, Michelle M. Epstein

**Affiliations:** 1 School of Environmental Sciences, University of East Anglia, Norwich, United Kingdom; 2 Earth System Physics Section, International Centre for Theoretical Physics, Trieste, Italy; 3 Laboratoire des sciences du climat et de l’environnement (LCSE), l’Institut Pierre Simon Laplace (IPSL), Centre d’Etudes Atomiques-Centre National de la Recherche Scientifique (CEA-CNRS) l’Université de Versailles Saint-Quentin (UVSQ), unité mixte de recherche (UMR) 8212, Gif sur Yvette, France; 4 Institut National de l’Environnement Industriel et des Risques, Parc technologique ALATA, Verneuil en Halatte, France; 5 Rothamsted Research, Harpenden, Hertfordshire, United Kingdom; 6 Department of Dermatology, Division of Immunology, Allergy and Infectious Diseases, Experimental Allergy, Medical University of Vienna, Vienna, Austria

## Abstract

**Background:**

Globally, pollen allergy is a major public health problem, but a fundamental unknown is the likely impact of climate change. To our knowledge, this is the first study to quantify the consequences of climate change upon pollen allergy in humans.

**Objectives:**

We produced quantitative estimates of the potential impact of climate change upon pollen allergy in humans, focusing upon common ragweed (*Ambrosia artemisiifolia*) in Europe.

**Methods:**

A process-based model estimated the change in ragweed’s range under climate change. A second model simulated current and future ragweed pollen levels. These findings were translated into health burdens using a dose–response curve generated from a systematic review and from current and future population data. Models considered two different suites of regional climate/pollen models, two greenhouse gas emissions scenarios [Representative Concentration Pathways (RCPs) 4.5 and 8.5], and three different plant invasion scenarios.

**Results:**

Our primary estimates indicated that sensitization to ragweed will more than double in Europe, from 33 to 77 million people, by 2041–2060. According to our projections, sensitization will increase in countries with an existing ragweed problem (e.g., Hungary, the Balkans), but the greatest proportional increases will occur where sensitization is uncommon (e.g., Germany, Poland, France). Higher pollen concentrations and a longer pollen season may also increase the severity of symptoms. Our model projections were driven predominantly by changes in climate (66%) but were also influenced by current trends in the spread of this invasive plant species. Assumptions about the rate at which ragweed spreads throughout Europe had a large influence upon the results.

**Conclusions:**

Our quantitative estimates indicate that ragweed pollen allergy will become a common health problem across Europe, expanding into areas where it is currently uncommon. Control of ragweed spread may be an important adaptation strategy in response to climate change.

**Citation:**

Lake IR, Jones NR, Agnew M, Goodess CM, Giorgi F, Hamaoui-Laguel L, Semenov MA, Solomon F, Storkey J, Vautard R, Epstein MM. 2017. Climate change and future pollen allergy in Europe. Environ Health Perspect 125:385–391; http://dx.doi.org/10.1289/EHP173

## Introduction

Climate change is likely to affect allergic disease (Smith et al. 2014), and the view of clinical experts is that these diseases will increase under climate change (Bielory et al. 2012) in part because of the impact on allergenic plant species (Shea et al. 2008). Impacts on allergens may be one of the most important consequences of climate change for human health (Beggs 2015). Climate change has already been suggested as one factor behind the increasing prevalence of allergic asthma (Beggs and Bambrick 2005). Pollens are a major cause of symptoms in people with allergic disease, but there is no quantitative assessment of how future climate change may affect the levels of pollen allergy in humans because the influence of climate change is complex (Reid and Gamble 2009; Smith et al. 2014). For example, an altered climate will affect the range of allergenic species as well as the timing and length of the pollen season, and elevated carbon dioxide (CO_2_) may increase plant productivity and pollen production (Beggs 2015). Climate change may also affect the release and atmospheric dispersion of pollen (Bielory et al. 2012). The overall impact will be alteration of pollen season timing and load, and hence, changes in exposure. Modeling all of these processes is needed to assess the consequences of climate change on pollen-related allergic disease. Previous studies (reviewed by Bielory et al. 2012) have only examined some of the processes along this pathway.

Allergic disease is a key public health problem that has increased rapidly in recent decades in both developed and developing countries, and it is now recognized as a major global epidemic (Pawankar 2014; Platts-Mills 2015). The economic burden of allergic disease is considerable. In 2007, the total cost of allergic disease to the United States was 19.7 billion USD, and estimates for the European Union range from 55 to 151 billion EUR (Zuberbier et al. 2014). In terms of specific allergic diseases, the World Health Organization (WHO) estimates that 400 million people in the world suffer from allergic rhinitis and 300 million from asthma (Bousquet and Khaltaev 2007). Within Europe, the prevalence of pollen allergy in the general population is estimated at 40% (D’Amato et al. 2007).

Here, we quantify the potential consequences of climate change on pollen allergy, focusing upon the annual herbaceous plant common ragweed (*Ambrosia artemisiifolia*) in Europe (henceforth referred to as ragweed). In Europe, ragweed is an introduced species in the middle of an ongoing invasion event (Storkey et al. 2014) and, therefore, represents a case of a human population being progressively exposed to a novel allergen. Ragweed is highly invasive; it thrives on disturbed land, with each plant producing ≤ 62,000 seeds per year. Ragweed is particularly harmful for public health because each plant produces a large amount of pollen (≤ 1 billion grains a year; Fumanal et al. 2007), and its allergenic potential is high (Taramarcaz et al. 2005). Unlike other types of pollen, ragweed pollen peaks in the late summer (Essl et al. 2015). In some parts of Europe, ragweed generates ~50% of the total pollen production. In the United States, where ragweed is native, > 26% of the population is sensitized to ragweed (Arbes et al. 2005). This study focuses upon ragweed sensitization as a health consequence. Sensitization occurs when the human immune system has synthesized antibodies against the pollen and reacts when reexposed, and it is a major risk factor for allergic diseases such as allergic rhinoconjunctivitis and asthma (Burbach et al. 2009).

## Methods

This research is the culmination of a large multidisciplinary European Commission funded project (Atopica®; FP7 grant agreement No. 282687). Specifically, for the present analysis, we integrated estimates of current and future ragweed pollen levels (previously developed within Atopica®) with published data on sensitization rates and population density. To estimate pollen levels, the Atopica® group first used a process-based model of weed growth, plant population dynamics, and competition to project the future expansion of ragweed’s range under different climate change scenarios (for details, see Storkey et al. 2014). These range results were then inputted into a system modeling plant invasion, pollen production, pollen release, and the atmospheric dispersion of pollen to simulate current (1986–2005) and future (2041–2060) ragweed pollen levels in Europe (for details, see Hamaoui-Laguel et al. 2015). To provide an estimate of uncertainty, these projections were produced for two different suites of regional climate/pollen models (henceforth, CHIMERE and WRF/RegCM; Hamaoui-Laguel et al. 2015). These projections differ in their driving global climate models and in their representation of dynamical and physical atmospheric processes. Both model suites used CMIP5 data (Taylor et al. 2012) to account for changing land use patterns. In addition, pollen levels were simulated under two alternative greenhouse gas concentration scenarios [Representative Concentration Pathway (RCP) 8.5, which assumes high emissions, and RCP4.5, which assumes moderate emissions] and three different ragweed plant invasion scenarios (slow, rapid, and reference rates of spread) (Hamaoui-Laguel et al. 2015). The outputs from these models were daily ragweed pollen levels for 50 km × 50 km grid cells across Europe. Two estimates of current levels were produced (based on CHIMERE or WRF/RegCM), as were 12 estimates of future levels (based on CHIMERE or WRF/RegCM, plus the 2 RCP and 3 plant invasion scenarios).

For the present analysis, these 14 estimates of current and future ragweed pollen were combined with health and population data to quantify their public health significance using ragweed sensitization rate (RSR) as a health consequence. Sensitization to ragweed is related to long-term pollen exposure; thus, these daily pollen outputs were aggregated to provide estimates, on a 50 km × 50 km grid, of average total season ragweed pollen.

To produce quantitative estimates of the potential impact of future ragweed pollen levels on RSRs, we first needed to generate a dose–response curve that would be representative of populations in locations with varying ragweed pollen levels in addition to characteristics that might confound the association between pollen levels and RSRs. Although we considered using estimates from an existing multicenter study [e.g., from the Atopica® project (Atopica® 2014), the GA^2^LEN study (Heinzerling et al. 2009), or the European Community Respiratory Health Survey (ECRHS) (Bousquet et al. 2007)], none of these was considered suitable as they either did not sample across a wide range of ragweed pollen levels, focused upon a subset of the population, or were restricted to patients with existing allergic disease.

We instead performed a systematic review of Web of Science, Medline, BIOSIS, the Cochrane library, OpenGrey, and Google Scholar to generate a pooled estimate of the dose–response curve between ragweed and RSR based on all available studies that met a clearly established set of criteria. We used the following major search terms as Medical Subject Headings (MeSH) and all-field text words, as appropriate: immunology, pollen, aeroallergen, allergy, allergens, atopy, sensitize (or sensitise), sensitized (or sensitised), sensitization (or sensitisation), hypersensitivity, skin test, and Immunoglobulin E. The initial search identified 1,923 potentially relevant papers (see Figure S1). A first filter of the search results (see Table S1) retained 50 papers that included estimates of RSRs for human populations within Europe (RSRs for 144 locations in total). Two authors (M.A. and I.L.) independently screened the remaining papers and extracted the following study information using standardized forms: location (country, place name), time period, sample characteristics (number, age, and population-based or allergy patients), the reactivity marker used (e.g., skin prick test, specific ragweed Immunoglobulin E), and the RSR. We then further excluded 15 papers that did not clearly report the sensitivity to ragweed alone, that involved a highly restricted sample population (e.g., institutionalized elderly, weed-sensitive allergy patients, patients with symptoms restricted to the ragweed season), that were undertaken more than two decades ago (i.e., pre-1993), or that had a small sample size (< 50 individuals). Finally, for studies that reported multiple RSRs at a single location (such as a series of annual sensitization observations, observations over several time periods, or observations for more than one age cohort) we selected a single RSR value representing the baseline period and the general population. Ultimately, the review comprised 66 location-specific RSRs from 35 studies and 20 European countries (see Table S2).

**Figure 1:**
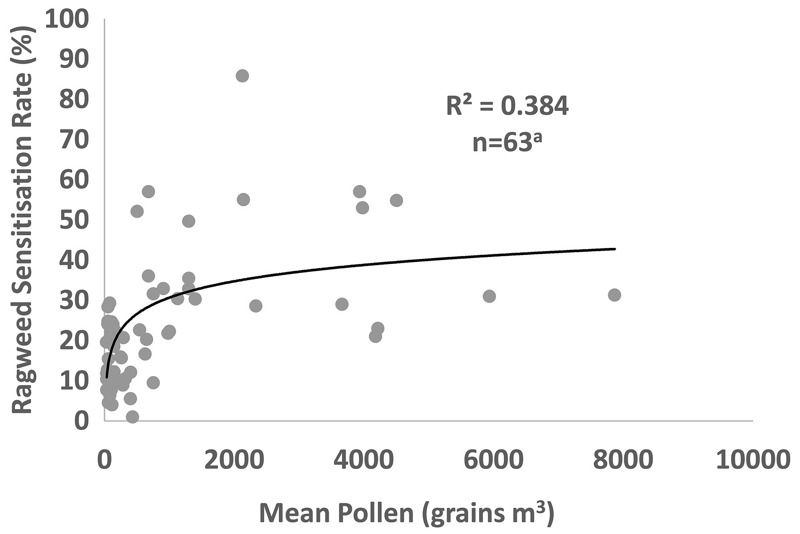
The relationship between ragweed sensitization rate (percent) and mean pollen level (grains per cubic meter), adjusting for study population. The solid line represents the best fit relationship (natural log). ***^a^***3 locations excluded owing to very low baseline pollen levels (< 10 grains/m^3^/year).

Each location-specific RSR was matched (on geographic coordinates) to modeled average total season ragweed pollen levels for the baseline period (1986–2005) reported by Hamaoui-Laguel et al. (2015). We then used a generalized linear model to estimate the association between the natural log–transformed mean pollen level (ln-mean pollen) at each location and the corresponding RSR for that location, adjusting for the study population type (general population, i.e., a random sample of individuals, or atopic population, e.g., allergy clinic patients). Although we considered adjusting for other factors known to affect RSR (e.g., skin prick test vs. allergen-specific IgE, atopic characteristics, and population characteristics such as age and sex), relevant data were often missing. The final model included 63 location-specific RSRs after excluding 3 locations with very low baseline pollen levels (< 10 grains/m^3^/year). The resulting model coefficient for ln-mean pollen was 5.85 [standard error (SE) 1.10, *p*-value < 0.001; 95% confidence interval (CI): 3.66, 8.04] with an adjusted *R* ^2^ = 0.384. The coefficient for study type (atopic vs. general population) was 9.35 (SE 3.70, *p*-value 0.014; 95% CI: 1.94, 16.76). A plot of the association between mean pollen counts and RSR, adjusted for study population type, is presented in Figure 1.

We then used the model coefficient and the estimated ln-mean pollen count in each 50 km × 50 km grid cell to estimate an RSR for each grid cell. This operation was performed on our 2 current and 12 future estimates of ragweed pollen across Europe. We combined the gridded RSR data with NUTS3 boundaries (subdivisions of the 28 EU countries into regions of 150,000–800,000 residents) and population data, to estimate the number of sensitized individuals at the NUTS3 level. These data were aggregated to the NUTS2 level (regions with 0.8–3 million residents) because many NUTS3 areas are smaller in size than the 50 km × 50 km grid cells. NUTS data were sourced from the Statistical Office of the European Union (Eurostat). For 12 non-EU European countries, we used boundaries and population data from the Global Administrative Areas database (DIVA-GIS 2015). Projections of population change were obtained from the World Bank databank (World Bank 2015) and applied equally to all NUTS2 areas within a country to estimate population counts for 2041–2060.

Burbach et al. (2009) indicated that only a proportion of patients sensitized to ragweed experience symptoms and presented estimates of clinically relevant sensitization rates for ragweed in different European countries. These estimates were obtained and were applied at the country level to the numbers of ragweed-sensitized individuals.

To estimate changes in the severity of ragweed allergy symptoms for sensitized individuals and the time period over which the symptoms are experienced, we also generated monthly maps of estimated ragweed pollen counts over the pollen season.

## Results

The pollen RSR dose–response function was applied to the 14 ragweed pollen maps on a 50 km × 50 km grid, and the corresponding population sensitized to ragweed at the NUTS2 level was mapped and tabulated at European and country levels. Initially focusing upon the reference plant invasion scenario, 6 RSR maps and country-level data on the sensitized population were produced (2 current + 4 future). These differed according to the regional climate/pollen model (CHIMERE or WRF/RegCM), time period (baseline or future), and RCP scenario used. These maps are presented in Figure S2 and Table S3. Estimates of the sensitized population based on airborne pollen levels generated using the WRF/RegCM model suite were 27–39% higher than corresponding estimates based on the CHIMERE model suite for both the baseline period and the future period. A comparison of the spatial patterns of current and future sensitization indicated that these model divergences were greatest in north and western Europe (Germany, Belgium, the Netherlands, and France), with models generated using the WRF/RegCM model suite indicating a greater sensitized population because of the higher levels of pollen in these locations. These results also indicated that the choice of RCP scenario makes little difference to the results by 2041–2060, irrespective of model suite; the sensitized populations differed by only ~5%. To provide an indication of the uncertainty, Table S3 also provides 95% confidence intervals (CIs) based upon the pollen RSR dose–response relationship. These indicate that there is relatively large uncertainty regarding the current and future sensitized population, a consequence of the divergent studies presented in Figure 1.

Here, the two model suites are considered to be equally plausible because they both show a similar performance in simulating pollen amounts compared with limited available observations (Hamaoui-Laguel et al. 2015). Therefore, the estimated population affected was averaged across the two model suites for all further analysis. However, in the numerical results, the data for both model suites are additionally reported in square brackets as an indicator of uncertainty; the following format is used: [CHIMERE, WRF/RegCM]. All subsequent results are presented for RCP4.5 (i.e., a moderate degree of climate change) for the sake of simplicity, bearing in mind that the numbers of sensitized individuals in the future are very similar between RCP4.5 and RCP8.5.

**Figure 2:**
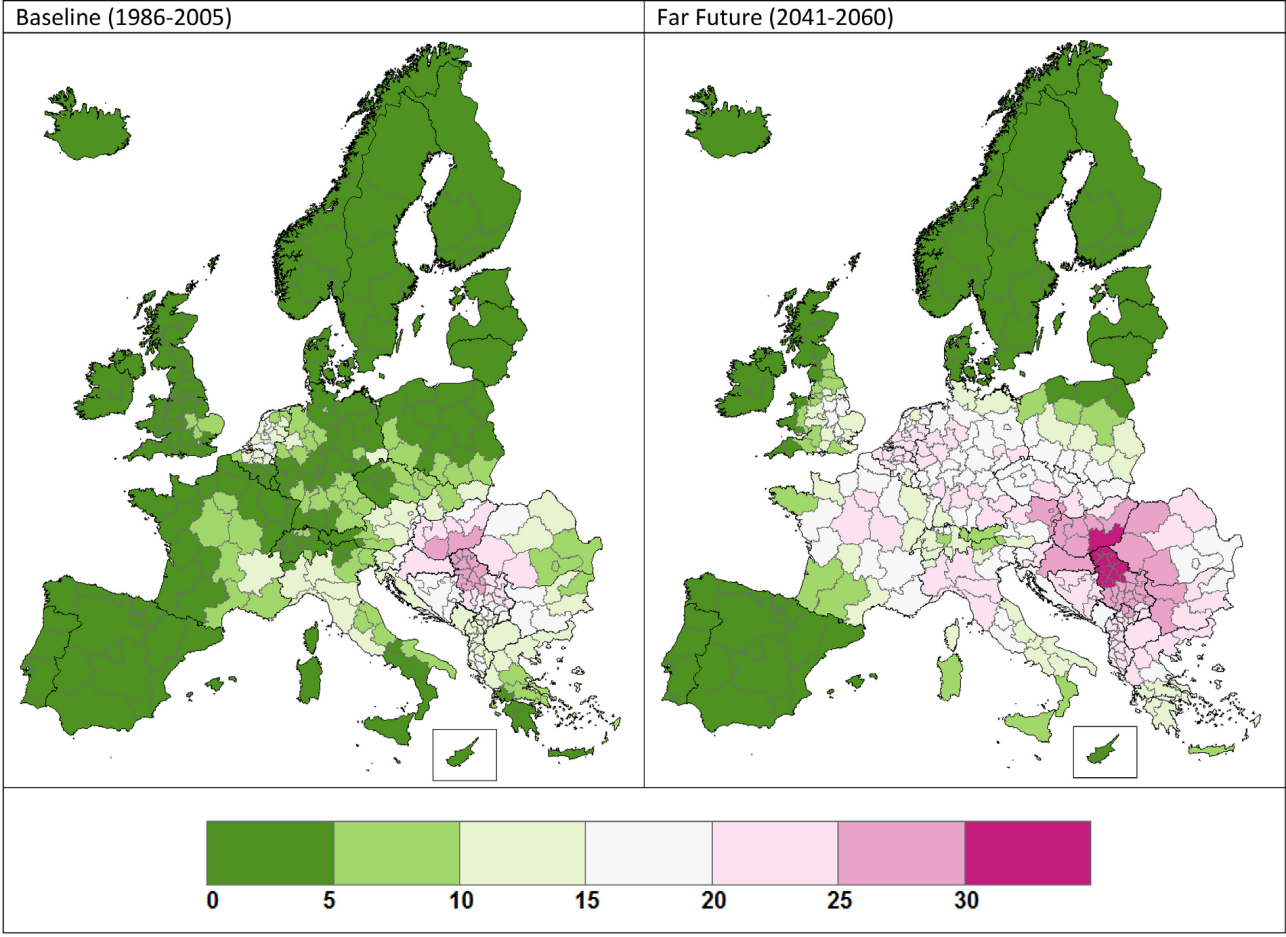
Percentage of population sensitized to ragweed pollen at baseline and in the far future; averaged results for WRF/RegCM and CHIMERE, RCP4.5, and reference invasion scenario. © EuroGeographics for the administrative boundaries.

Our best estimates of the current and future population sensitized to ragweed from the pollen RSR dose–response relationship shown in Figure 1 are presented in Figure 2. The sensitized population numbers are presented in Table 1 at the country level and are summed for EU28 and non-EU28 countries and for Europe as a whole. Overall, our estimates indicate that under the RCP4.5 emissions scenario and the reference ragweed invasion scenario, the number of people sensitized to ragweed in Europe would increase from approximately 33 million (27 million and 38 million based on CHIMERE and WRF/RegCM, respectively) at baseline to 77 million (68 million and 86 million based on CHIMERE and WRF/RegCM, respectively) in 2041–2060 owing to higher pollen counts affecting a larger spatial area. Sensitization is projected to increase in countries with an existing ragweed problem, such as Romania and Italy, partly as a result of increased pollen production by established plant populations; however, the greatest proportional increases are likely to be in areas where ragweed sensitization is currently relatively uncommon, such as Germany, France, and Poland. By 2041–2060, sensitization to ragweed will be widespread across the whole of Europe except for Scandinavia, the Baltic States, most of Spain, Portugal, and Ireland.

**Table 1:** Current and future populations sensitized to ragweed pollen for three different plant invasion scenarios. Data are average of the CHIMERE and WRF/RegCM model suites for Representative Concentration Pathway (RCP) 4.5 [CHIMERE and WRF/RegCM values given in brackets].

Country	Population sensitized in thousands [CHIMERE, WRF/RegCM values]			
	Baseline	2041–2060		
		Reference ragweed invasion scenario	Slow ragweed invasion scenario	Rapid ragweed invasion scenario
Austria	890 [868, 912]	1,749 [1,636, 1,863]	1,354 [1,282, 1,427]	2,061 [1,954, 2,169]
Belgium	923 [732, 1,115]	2,364 [2,143, 2,585]	1,616 [1,452, 1,780]	2,767 [2,519, 3,015]
Bulgaria	1,150 [1,135, 1,165]	1,763 [1,614, 1,912]	1,442 [1,350, 1,535]	2,066 [1,889, 2,243]
Croatia	873 [804, 943]	1,098 [1,041, 1,156]	1,037 [973, 1,102]	1,169 [1,124, 1,214]
Cyprus	7 [0, 15]	36 [1, 71]	15 [0, 31]	77 [33, 120]
Czech Republic	487 [409, 565]	1,943 [1,756, 2,130]	1,150 [1,054, 1,246]	2,844 [2,737, 2,950]
Denmark	0 [0, 0]	96 [29, 163]	3 [0, 7]	533 [426, 640]
Estonia	0 [0, 0]	0 [0, 0]	0 [0, 0]	5 [3, 7]
Finland	0 [0, 0]	0 [0, 0]	0 [0, 0]	0 [0, 0]
France	3,233 [2,256, 4,210]	10,716 [8,849, 12,582]	5,989 [4,480, 7,498]	15,646 [14,066, 17,225]
Germany	4,688 [2,282, 7,095]	15,689 [13,337, 18,041]	9,321 [6,882, 11,759]	20,928 [19,129, 22,727]
Greece	831 [487, 1,176]	1,764 [1,341, 2,188]	1,284 [911, 1,658]	2,320 [1,908, 2,732]
Hungary	2,289 [2,668, 1,910]	2,899 [3,069, 2,729]	2,721 [2,979, 2,464]	3,006 [3,098, 2,914]
Ireland	0 [0, 0]	4 [0, 8]	0 [0, 0]	82 [0, 164]
Italy	4,786 [4,097, 5,474]	10,110 [9,563, 10,656]	7,480 [6,846, 8,115]	13,450 [13,079, 13,821]
Latvia	0 [0, 0]	0 [0, 0]	0 [0, 0]	77 [78, 75]
Lithuania	0 [0, 0]	6 [11, 2]	0 [0, 0]	224 [245, 204]
Luxembourg	15 [0, 31]	78 [57, 98]	33 [8, 58]	124 [106, 141]
Malta	0 [0, 0]	28 [18, 38]	12 [5, 19]	49 [38, 60]
Netherlands	2,224 [1,300, 3,148]	3,489 [2,863, 4,115]	2,894 [2,135, 3,654]	3,862 [3,374, 4,350]
Poland	1,123 [1,251, 994]	4,397 [4,175, 4,619]	2,437 [2,467, 2,408]	8,733 [8,382, 9,084]
Portugal	0 [0, 0]	0 [0, 0]	0 [0, 0]	356 [0, 712]
Romania	3,097 [3,045, 3,148]	4,772 [4,473, 5,072]	4,016 [3,796, 4,237]	5,500 [5,062, 5,938]
Slovakia	626 [790, 462]	1,160 [1,221, 1,100]	895 [1,004, 786]	1,438 [1,419, 1,457]
Slovenia	304 [281, 327]	424 [397, 451]	385 [364, 406]	470 [442, 497]
Spain	21 [0, 42]	447 [35, 858]	137 [1, 272]	2,670 [939, 4,401]
Sweden	0 [0, 0]	13 [1, 25]	0 [0, 0]	153 [114, 192]
United Kingdom	1,196 [1,008, 1,384]	6,173 [5,113, 7,232]	2,631 [1,988, 3,273]	10,023 [9,270, 10,776]
**Sum EU28**	**28,764 [23,413, 34,116]**	**71,218 [62,743, 79,693]**	**46,855 [39,975, 53,736]**	**100,631 [91,434, 109,828]**
Albania	394 [400, 388]	626 [580, 673]	513 [496, 529]	784 [727, 840]
Andorra	0 [0, 0]	2 [0, 5]	0 [0, 1]	9 [7, 11]
Bosnia and Herzegovina	703 [682, 724]	914 [869, 958]	855 [819, 890]	980 [937, 1,023]
Iceland	0 [0, 0]	0 [0, 0]	0 [0, 0]	0 [0, 0]
Kosovo	259 [196, 323]	429 [391, 468]	347 [287, 407]	498 [492, 504]
Liechtenstein	1 [0, 1]	3 [1, 4]	1 [0, 3]	4 [3, 6]
FYR Macedonia	300 [291, 308]	486 [449, 524]	395 [369, 420]	584 [562, 605]
Montenegro	78 [56, 101]	124 [101, 146]	105 [81, 129]	148 [131, 164]
Norway	0 [0, 0]	0 [0, 0]	0 [0, 0]	4 [0, 7]
San Marino	2 [2, 2]	5 [5, 4]	4 [4, 4]	6 [6, 6]
Serbia	1,708 [1,731, 1,685]	2,073 [2,042, 2,105]	1,964 [1,928, 1,999]	2,140 [2,129, 2,151]
Switzerland	376 [135, 618]	954 [650, 1,257]	650 [346, 954]	1,498 [1,222, 1,774]
**Sum non-EU28**	**3,822 [3,493, 4,151]**	**5,615 [5,087, 6,144]**	**4,833 [4,331, 5,335]**	**6,654 [6,216, 7,091]**
**Sum Europe**	**32,586 [26,905, 38,266]**	**76,833 [67,829, 85,837]**	**51,688 [44,305, 59,071]**	**107,285 [97,650, 116,919]**

Table 1 also examines the impact of ragweed invasion scenario upon sensitization rates. Under the reference plant invasion scenario, the population sensitized to ragweed at the European level is estimated to increase from 33 million (range: 27 million–38 million) to 77 million (range: 68 million–86 million) by 2041–2060. In comparison, a slow plant invasion scenario reduces the projected future value to approximately 52 million (range: 44 million–59 million), whereas a rapid plant invasion scenario increases the projected value to approximately 107 million (range: 98 million–117 million).

The sensitization rates presented in Table 1 were converted into estimates of clinically relevant sensitization rates using data from Burbach et al. (2009). These results are presented in Table S4, indicating that compared with the population sensitized to ragweed, the population clinically sensitized to ragweed in Europe is approximately 25% lower for both the baseline and 2041–2060. Table S4 also shows that future changes in the European population base do not greatly affect our projections, but they suggest smaller impacts in countries with decreasing populations such as Germany, Poland, and Romania, and accentuated impacts in countries with increasing populations (e.g., France, the United Kingdom).

**Figure 3:**
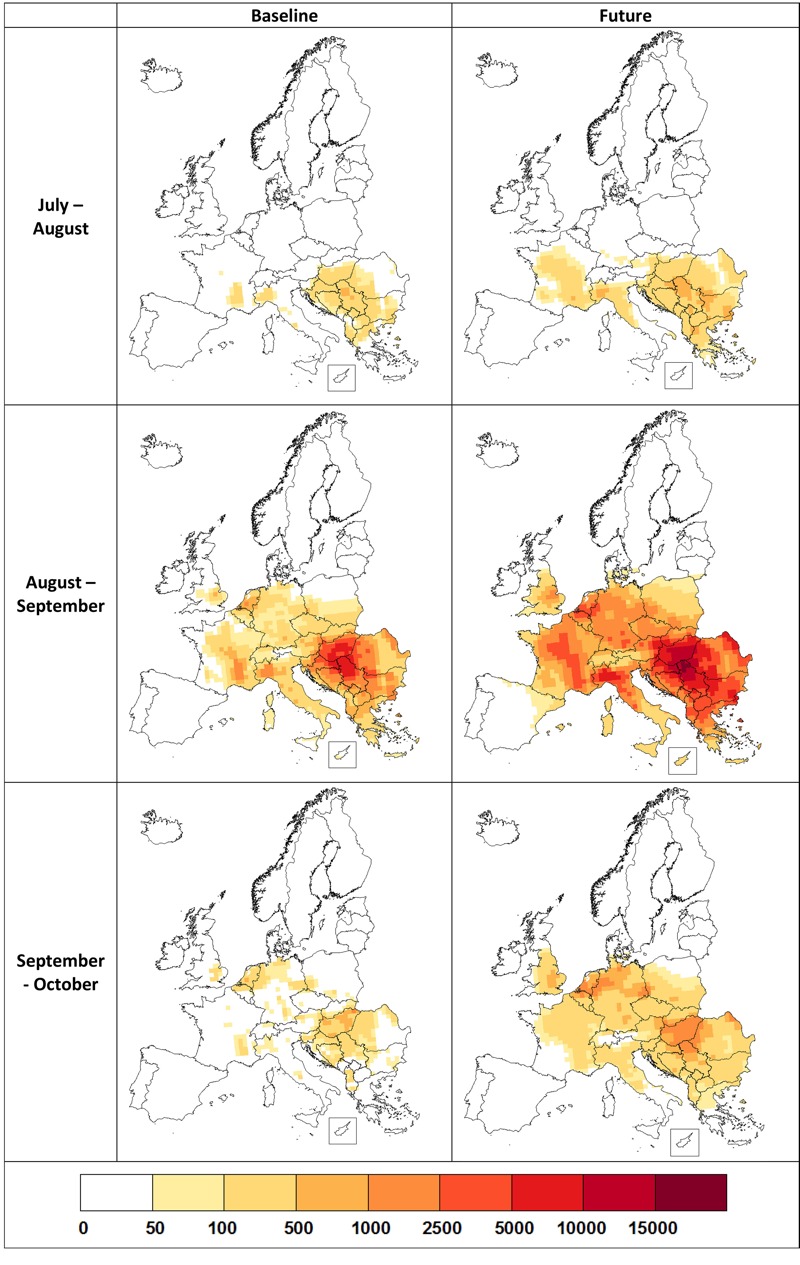
Monthly current (baseline) and future (2041–2060) ragweed pollen counts (grains per cubic meter) across Europe. Data are the average of the CHIMERE and WRF/RegCM model suites for RCP4.5 and a reference plant invasion scenario. © EuroGeographics for the administrative boundaries.

To estimate the potential impacts of future climate-related changes on allergy symptoms for individuals sensitized to ragweed, maps of average total season pollen (mid-July–mid-October) subdivided into monthly periods were produced and are displayed in Figure 3. These estimates indicate that particularly across France and northwest Italy, airborne pollen is likely to be present much earlier in the season (mid-July–mid-August) because of accelerated plant development. In the peak pollen months (mid-August–mid-September), more ragweed pollen is likely to be present across Europe, with the greatest increases occurring away from current pollen hotspots. Our projections suggest that pollen will persist in the air across most of Europe in the mid-September to mid-October period, likely as a result of delayed frosts (Storkey et al. 2014).

## Discussion

It has been argued that climate change is likely to affect pollen-related allergy (Bielory et al. 2012). To our knowledge, this is the first study to fully model the potential impacts of climate change on ragweed plant distribution, plant productivity, and pollen production and dispersal, as well as the resulting impacts on pollen concentrations and allergic sensitization.

We estimate that across Europe, sensitization to ragweed is likely to more than double by 2041–2060 and that populations across most of Europe are likely to be affected. Our projections indicate that sensitization will continue to increase in countries with an existing ragweed problem, but the greatest proportional increases will be in areas where ragweed sensitization is currently relatively uncommon. Much of the projected change is due to the expected northward expansion of ragweed, consistent with the expansion already observed in the United States (Ziska et al. 2011). Our estimates indicate that ragweed-sensitized individuals may experience more severe symptoms because of increased ragweed pollen concentrations and a longer pollen season lasting into September and October across much of Europe. These projected changes are predominantly related to climate and associated land-use change (66%; Hamaoui-Laguel et al. 2015) but also include the dispersal of this alien plant through Europe even without climate change.

One striking feature of the results is the large influence of the plant invasion scenario. The reference plant invasion scenario uses the common assumption that the flux of seeds is inversely proportional to the square of the distance (Hamaoui-Laguel et al. 2015). The slow and rapid plant invasion scenarios were generated by altering the proportionality coefficient based upon the ranges reported in previous research (Richter et al. 2013). The slow plant invasion scenario assumes a limited expansion in the range of ragweed, and this scenario more than halves the overall estimated increase in sensitization to ragweed projected by 2041–2060 under RCP4.5. Thus, it strongly suggests that control of ragweed is important for public health and as an adaptation strategy against the impacts of climate change. However, control of existing plants is difficult owing to ragweed’s long-lived seeds, to its ability to evolve herbicide resistance, and to its capacity to resprout following cutting (Brewer and Oliver 2009). Ragweed thrives on regular land disturbance; hence, management of land is key to its control (Storkey et al. 2014). Controlling long-distance seed dispersal is also important for preventing plant spread, which is predominantly associated with human activity. Therefore, controls over contaminated seed and monitoring areas prone to ragweed invasion are key elements to limiting spread (Bullock 2010).

We also examined the impact of different Representative Concentration Pathways (RCP 4.5 and 8.5) and highlighted that these pathways make little difference to the numbers of individuals sensitized to ragweed. This outcome is likely to be a result of saturation of the CO_2_ fertilization effect at higher concentrations; it may also result from the relatively similar climate between the two RCPs within the relatively short future time frame of our analysis. Projected changes to the population numbers and distribution across Europe had a relatively minor influence on our results.

This study emphasizes the multiple steps required to model the impact of climate change on pollen allergy. There are assumptions and uncertainties associated with each step of the process, and as far as possible, we have been transparent about them and about their impacts upon the results. We compared two regional climate model suites (WRF/RegCM, CHIMERE) that differ in atmospheric processing, in pollen modeling, and in the driving global climate model, and we found responses in the same direction, although with substantial differences in magnitude. This finding highlights the need for multimodel approaches to the problem of future pollen simulation. Our research was highly sensitive to the assumptions concerning the plant invasion scenarios, which emphasizes the importance of dispersal control [e.g., the measures highlighted by Bullock (2010)] as an effective tool to minimize ragweed allergy in the future.

A notable element of uncertainty is the pollen/RSR dose–response relationship (adjusted *R* ^2^ = 38.4%). This low value is a function of the limited number of studies reporting sensitization to ragweed and the lack of standardization across studies. We have also assumed that the dose–response relationship between pollen and allergy is identical across Europe, whereas differences in factors such as genetic predisposition may lead to a differential impact across the continent.

In addition to climate change, plant invasion and population change, other factors may affect ragweed allergy moving into the future. By 2041–2060, levels of ozone air pollution across Europe are likely to decrease (Colette et al. 2012), potentially suppressing the allergenicity of ragweed pollen (Pasqualini et al. 2011). Conversely, ragweed pollen allergenicity may be elevated through higher atmospheric CO_2_ levels and increasing drought (El Kelish et al. 2014; Singer et al. 2005). Inclusion of changing allergenicity is a priority for future research. By 2041–2060, the median age of the European population is projected to increase from 38 to 52 years (World Bank 2015), and ragweed allergy is more difficult to treat in aged populations owing to greater difficulty in diagnosis and limited treatment options because of comorbidities and ongoing medication use (Cardona et al. 2011). Appropriate management and use of medication can significantly improve allergy symptoms (Pawankar 2014). Such management can be economically beneficial, and appropriate therapy for allergic diseases can be 5% of the cost of untreated disease (Zuberbier et al. 2014). Therefore, the overall impact of increasing ragweed allergy will be influenced by the adaptation capacities of individuals and by those of healthcare systems across Europe. Ebi (2014) argued that the capacity of healthcare systems to adapt to climate change will depend upon the development pathways taken by individual countries. Pathways leading to increased inequalities and fragmentation in society present most of the challenges to adaptation (Ebi 2014) and hence to the potential problem of increasing ragweed allergy.

## Conclusions

Our projections indicate that ragweed pollen allergy will become a common health problem across much of Europe and that sensitization to ragweed will more than double, increasing from the current total of 33 million to 77 million people by 2041–2060. According to our projections, sensitization will increase in countries with an existing ragweed problem (e.g., Hungary, the Balkans) but the greatest proportional increases are projected for countries where sensitization is now relatively uncommon (e.g., Germany, Poland, France). Our estimates also indicate that sensitized individuals may experience more severe symptoms as a consequence of higher ragweed pollen levels and an extended pollen season that will last into September and October across much of Europe. Our projections are primarily driven by assumptions regarding climate change (66%) but also reflect current trends in the spread of this invasive plant species across Europe. The projected health consequences are highly dependent upon the rate at which ragweed spreads, which is strongly related to control measures against the spread of this plant species (Bullock 2010). This relationship emphasizes that control of ragweed spread is essential for public health and as an adaptation strategy in response to climate change.

To our knowledge, this is the first study to model the future impacts of climate change on plant distribution, plant life cycles, and pollen production and dispersal, as well as their subsequent impacts on pollen concentrations and allergy. Climate change consequences will not be restricted to ragweed, and a recent review has highlighted a range of other pollen-producing species that may be affected (Beggs 2015). Our methods provide a framework for other studies investigating the impacts of climate change on pollen allergy for these other species.

## Supplemental Material

(412 KB) PDF

## References

[r1] Arbes SJ, Gergen PJ, Elliott L, Zeldin DC (2005). Prevalences of positive skin test responses to 10 common allergens in the US population: results from the Third National Health and Nutrition Examination Survey.. J Allergy Clin Immunol.

[r2] ATOPICA (Atopic Diseases in Changing Climate, Land Use and Air Quality) (2014). Atopica Final Report.. http://cordis.europa.eu/result/rcn/171069_en.html.

[r3] Beggs PJ (2015). Environmental allergens: from asthma to hay fever and beyond.. Curr Clim Change Rep.

[r4] Beggs PJ Bambrick HJ 2005 Is the global rise of asthma an early impact of anthropogenic climate change? Environ Health Perspect 113 915 919, doi:10.1289/ehp.7724 16079058 PMC1280328

[r5] Bielory L, Lyons K, Goldberg R (2012). Climate change and allergic disease.. Curr Allergy Asthma Rep.

[r6] Bousquet J, Khaltaev N, eds (2007). *Global Surveillance, Prevention and Control of Chronic Respiratory Diseases: A Comprehensive Approach*..

[r7] Bousquet PJ, Chinn S, Janson C, Kogevinas M, Burney P, Jarvis D (2007). Geographical variation in the prevalence of positive skin tests to environmental aeroallergens in the European Community Respiratory Health Survey I.. Allergy.

[r8] Brewer CE, Oliver LR (2009). Confirmation and resistance mechanisms in glyphosate-resistant common ragweed (*Ambrosia artemisiifolia*) in Arkansas.. Weed Sci.

[r9] Bullock J (2010). *Assessing and Controlling the Spread and the Effects of Common Ragweed in Europe*. ENV.B2/ETU/2010/0037.. http://ec.europa.eu/environment/nature/invasivealien/docs/Final_Final_Report.pdf.

[r10] Burbach GJ, Heinzerling LM, Edenharter G, Bachert C, Bindslev-Jensen C, Bonini S (2009). GA^2^LEN skin test study II: clinical relevance of inhalant allergen sensitizations in Europe.. Allergy.

[r11] Cardona V Guilarte M Luengo O Labrador-Horrillo M Sala-Cunill A Garriga T 2011 Allergic diseases in the elderly. Clin Transl Allergy 1 11, doi:10.1186/2045-7022-1-11 22409889 PMC3339328

[r12] Colette A, Granier C, Hodnebrog Ø, Jakobs H, Maurizi A, Nyiri A (2012). Future air quality in Europe: a multi-model assessment of projected exposure to ozone.. Atmos Chem Phys.

[r13] D’Amato G, Cecchi L, Bonini S, Nunes C, Annesi-Maesano I, Behrendt H (2007). Allergenic pollen and pollen allergy in Europe.. Allergy.

[r14] DIVA-GIS (2015). Download Data by Country. GADM Version 1.0.. http://www.diva-gis.org/gdata.

[r15] Ebi KL (2014). Health in the new scenarios for climate change research.. Int J Environ Res Public Health.

[r16] El Kelish A Zhao F Heller W Durner J Winkler JB Behrendt H 2014 Ragweed (*Ambrosia artemisiifolia*) pollen allergenicity: SuperSAGE transcriptomic analysis upon elevated CO_2_ and drought stress. BMC Plant Biology 14 176, doi:10.1186/1471-2229-14-176 24972689 PMC4084800

[r17] Essl F, Biró K, Brandes D, Broennimann O, Bullock JM, Chapman DS (2015). Biological flora of the British Isles: *Ambrosia artemisiifolia*.. J Ecol.

[r18] Fumanal B, Chauvel B, Bretagnolle F (2007). Estimation of pollen and seed production of common ragweed in France.. Ann Agric Environ Med.

[r19] Hamaoui-Laguel L, Vautard R, Liu L, Solmon F, Viovy N, Khvorosthyanov D (2015). Effects of climate change and seed dispersal on airborne ragweed pollen loads in Europe [Letter].. Nat Clim Chang.

[r20] Heinzerling LM, Burbach GJ, Edenharter G, Bachert C, Bindslev-Jensen C, Bonini S (2009). GA^2^LEN skin test study I: GA^2^LEN harmonization of skin prick testing: novel sensitization patterns for inhalant allergens in Europe.. Allergy.

[r21] Pasqualini S, Tedeschini E, Frenguelli G, Wopfner N, Ferreira F, D’Amato G (2011). Ozone affects pollen viability and NAD(P)H oxidase release from *Ambrosia artemisiifolia* pollen.. Environ Pollut.

[r22] Pawankar R 2014 Allergic diseases and asthma: a global public health concern and a call to action. World Allergy Organ J 7 12, doi:10.1186/1939-4551-7-12 24940476 PMC4045871

[r23] Platts-Mills TAE (2015). The allergy epidemics: 1870–2010.. J Allergy Clin Immunol.

[r24] Reid CE, Gamble JL (2009). Aeroallergens, allergic disease, and climate change: impacts and adaptation.. Ecohealth.

[r25] Richter R, Dullinger S, Essl F, Leitner M, Vogl G (2013). How to account for habitat suitability in weed management programmes?. Biol Invasions.

[r26] Shea KM, Truckner RT, Weber RW, Peden DB (2008). Climate change and allergic disease.. J Allergy Clin Immunol.

[r27] Singer BD, Ziska LH, Frenz DA, Gebhard DE, Straka JG (2005). Increasing Amb a 1 content in common ragweed (*Ambrosia artemisiifolia*) pollen as a function of rising atmospheric CO_2_ concentration.. Funct Plant Biol.

[r28] Smith KR, Woodward A, Campbell-Lendrum D, Chadee DD, Honda Y, Liu Q, et al (2014). Human health: impacts, adaptation, and co-benefits. In: *Climate Change 2014: Impacts, Adaptation, and Vulnerability Part A: Global and Sectoral Aspects Contribution of Working Group II to the Fifth Assessment Report of the Intergovernmental Panel on Climate Change*. Field CB, Barros VR, Dokken DJ, Mach KJ, Mastrandrea MD, Bilir TE, et al., eds..

[r29] Storkey J Stratonovitch P Chapman DS Vidotto F Semenov MA 2014 A process-based approach to predicting the effect of climate change on the distribution of an invasive allergenic plant in Europe. PLoS One 9 e88156, doi:10.1371/journal.pone.0088156 24533071 PMC3922760

[r30] Taramarcaz P, Lambelet C, Clot B, Keimer C, Hauser C (2005). Ragweed (Ambrosia) progression and its health risks: will Switzerland resist this invasion?. Swiss Med Wkly.

[r31] Taylor KE, Stouffer RJ, Meehl GA (2012). An overview of CMIP5 and the experiment design.. Bull Am Meteorol Soc.

[r32] World Bank (2015). Health, nutrition and population statistics.. http://databank.worldbank.org/data/reports.aspx?source=health-nutrition-and-population-statistics.

[r33] Ziska L, Knowlton K, Rogers C, Dalan D, Tierney N, Elder MA (2011). Recent warming by latitude associated with increased length of ragweed pollen season in central North America.. Proc Natl Acad Sci U S A.

[r34] Zuberbier T, Lötvall J, Simoens S, Subramanian SV, Church MK (2014). Economic burden of inadequate management of allergic diseases in the European Union: a GA^2^LEN review.. Allergy.

