# Supplementary material for: Climate Change and Future Pollen Allergy in Europe
Source: Environ Health Perspect. 2016 Aug 24;125(3):385–91. doi: 10.1289/EHP173 (PMC5332176; doi:10.1289/EHP173)
Supplement: (412 KB) PDF [file EHP173.s001.acco.pdf]

Environ Health Perspect
DOI: 10.1289/EHP173

Note to readers with disabilities: *EHP* strives to ensure that all journal content is accessible to all readers. However, some figures and Supplemental Material published in *EHP* articles may not conform to 508 standards due to the complexity of the information being presented. If you need assistance accessing journal content, please contact ehp508@niehs.nih.gov. Our staff will work with you to assess and meet your accessibility needs within 3 working days.

Supplemental Material

Climate Change and Future Pollen Allergy in Europe

Iain R. Lake, Natalia R. Jones, Maureen Agnew, Clare M. Goodess, Filippo Giorgi, Lynda Hamaoui-Laguel, Mikhail A. Semenov, Fabien Solomon, Jonathan Storkey, Robert Vautard, and Michelle M. Epstein

Table of Contents

Figure S1: Process for selecting studies reporting sensitization to ragweed pollen
Table S1: Ragweed Sensitization Rate (RSR) exclusion criteria
Table S2: Ragweed sensitization rates included in the review and summary information collected
References
Figure S2: Baseline and future population (percentage) sensitized to ragweed pollen at the NUTS2 level for RCP4.5 and RCP8.5 and a reference plant invasion scenario. Data are presented separately for the CHIMERE and WRF/RegCM model suites.
Table S3: Population (in thousands) sensitized to ragweed for the current and future. Reference ragweed invasion scenario comparing RCP 4.5 and RCP 8.5, and 2 climate model suites (Chimere, WRF/RegCM) for 40 European countries; 95% confidence interval (CIs) shown in brackets.
Table S4: Current and future populations clinically sensitized to ragweed pollen. Changes due to population change are also presented. Data are average of the CHIMERE and WRF/RegCM model suites for RCP4.5 and a reference plant invasion scenario.

Figure S1: Process for selecting studies reporting sensitization to ragweed pollen

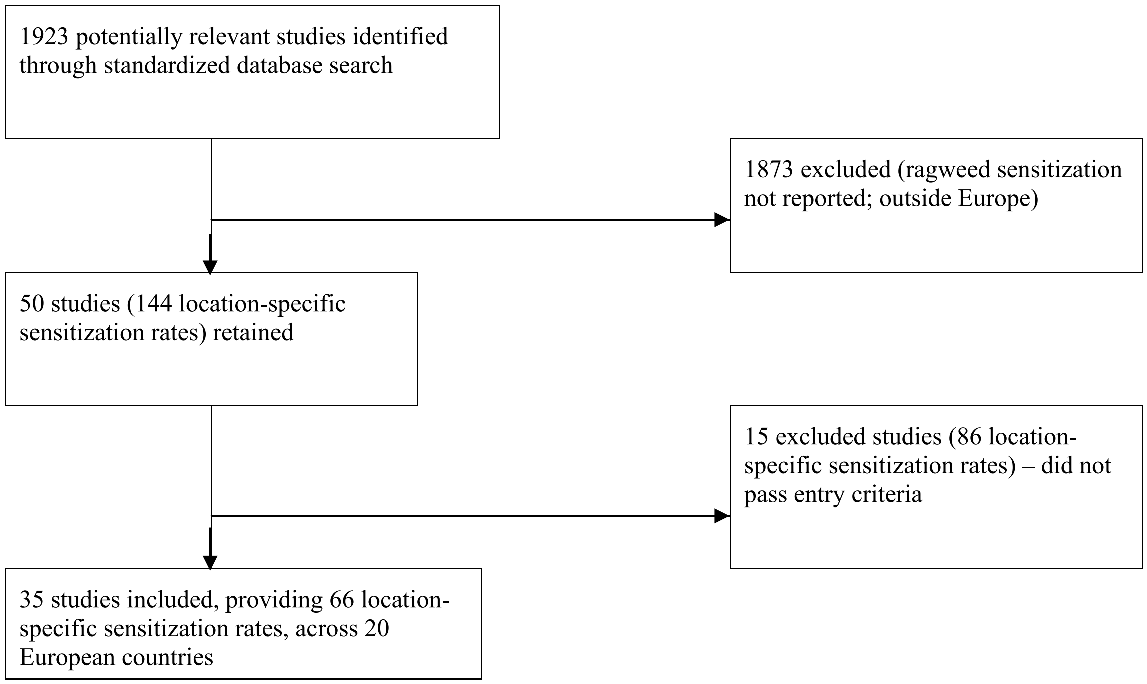

Table S1: Ragweed Sensitization Rate (RSR) exclusion criteria

**First filter:**
Ragweed sensitization is not reported in the study
The study has taken place outside Europe
**Second filter exclusion criteria:**
RSR is not clearly restricted to ragweed pollen alone but e.g., for ragweed and/or mug wort
RSR is reported for an unrepresentative or overly-focused sub group of the population (e.g. an elderly cohort living in an institution, weed-sensitive allergy patients, allergy patients only symptomatic during ragweed season, immigrants)
The sample size is < 50 individuals
The date of the study is pre 1993 (i.e., over 20 years before the start of the present project)

Table S2: Ragweed sensitization rates included in the review and summary information collected

Citation	Year(s) of data collection	Location of data collection: country, place name	Study size	Population characteristics (including age, allergy/atopic patients or general population)	Sensitization rate (%)	Reactivity marker‡
(Hemmer et al. 2011)	1997-2007	Austria, all regions	13719	Atopic patients diagnosed between 1997 and 2007	11.1	SPT
(Hemmer et al. 2011)	1997-2007	Austria, Alpine regions	13719	Atopic patients diagnosed between 1997 and 2007	5.4 (4.7-6.0)	SPT
(Hemmer et al. 2011)	1997-2007	Austria, Eastern lowlands adjoining Hungary	13719	Atopic patients diagnosed between 1997 and 2007	20.8	SPT
(Heinzerling et al. 2009)	2006–2007	Austria, Vienna	not given*	Standardized allergy patients (GA^2LEN)	8.5	SPT
(Jäger 2000)	1984-1997	Austria, Vienna	3510 (1993-97)	Allergy outpatients restricted to the period 1993-1997.	30.6	SPT + sp IGE
(Heinzerling et al. 2009)	2006–2007	Belgium, Ghent	not given*	Standardized allergy patients (GA^2LEN)	3.0	SPT
(Nikolov et al. 2013)	2011-2012	Bulgaria, Sofia	74	74 patients sensitized to grass and/or weed pollen	21.6	SPT
(Toth et al. 2011)	2003-2006	Croatia, Ivanic Grad	614	Patients referred to respiratory hospital for the first time for skin prick tests	51.1	SPT
(ATOPICA 2014)	2012-2014	Croatia, Ivanic Grad	157	Children (aged 2-13y), general population	19.1	SPT (≥ 2 mm)
(ATOPICA 2014)	2012-2014	Croatia, Osijek	582	Children (aged 2-13y), general population	17.5	SPT (≥ 2 mm)
(Toth et al. 2011)	2003-2006	Croatia, Samobor	493	Patients referred to respiratory hospital for the first time for skin prick tests	15.6	SPT
(ATOPICA 2014)	2012-2014	Croatia, Samobor	169	Children (aged 2-13y), general population	16.6	SPT (≥ 2 mm)
(ATOPICA 2014)	2012-2014	Croatia, Slavonski Brod	540	Children (aged 2-13y), general population	15.2	SPT (≥ 2 mm)
(ATOPICA 2014)	2012-2014	Croatia, Split	1180	Children (aged 2-13y), general population	1.9	SPT (≥ 2 mm)
(Cvitanovic et al. 2007)	2003	Croatia, Split-Dalmatia	320	320 patients from Split-Dalmatian County with seasonal rhinitis and	17.5	SPT

Citation	Year(s) of data collection	Location of data collection: country, place name	Study size	Population characteristics (including age, allergy/atopic patients or general population)	Sensitization rate (%)	Reactivity marker[‡]
				asthma symptoms		
(ATOPICA 2014)	2012-2014	Croatia, Velika Gorica	182	Children (aged 2-13y), general population	16.5	SPT (≥ 2 mm)
(ATOPICA 2014)	2012-2014	Croatia, Zadar	390	Children (aged 2-13y), general population	2.1	SPT (≥ 2 mm)
(ATOPICA 2014)	2012-2014	Croatia, Zagreb	816	Children (aged 2-13y), general population	14.8	SPT (≥ 2 mm)
(Toth et al. 2011)	2003-2006	Croatia, Zagreb	1085	Patients referred to respiratory hospital for the first time for skin prick tests. Mean of north and south Zagreb	34.5	SPT
(Peternel et al. 2008)	2004	Croatia, Zagreb city	750	Adult patients presenting for first time at respiratory clinic (aged 18-80y)	48.7	SPT
(Mehulić et al. 2011)	1991-1994; 1995-1999, 2000-2004	Croatia; Zagreb and surrounding areas	2577 (794, 814, 969)	Outpatient allergy clinic. Mean of three time periods	31.9	SPT / sp IGE
(Rybníček et al. 2000)	1995	Czech Republic, Brno	94	Children with pollen allergy (symptomatic)	22.0	SPT + sp IgE
(Rybníček et al. 2000)	1995-1997	Czech Republic, Brno	645	Adult allergic patients suggestive of pollen allergy	22.9	SPT + sp IgE
(Heinzerling et al. 2009)[a]	2006–2007	Denmark, Odense	not given*	Standardized allergy patients (GA^2LEN)	17.1	SPT
(Heinzerling et al. 2009) [a]	2006–2007	Finland, Helsinki	not given*	Standardized allergy patients (GA^2LEN)	2.3	SPT
(Heinzerling et al. 2009)	2006–2007	France, Montpellier	not given*	Standardized allergy patients (GA^2LEN)	9.0	SPT
(Deloraine and Rouvière 2000)	1999	France, Rhone-Alps (Lyon and surrounding areas)	4988	Adults (general population) contacted by telephone survey	8.5	questionnaire
(Gabrio et al.	2004-2009	Germany, Baden-Württemberg	2678	10 year old (Grade 4) population	14.6	sp IgE + SPT

Citation	Year(s) of data collection	Location of data collection: country, place name	Study size	Population characteristics (including age, allergy/atopic patients or general population)	Sensitization rate (%)	Reactivity marker[‡]
2010)				study		
(Gabrio et al. 2010)	2008-2009	Germany, Baden-Württemberg	1134	Adults (general population)	11.0	sp IgE + SPT
(Heinzerling et al. 2009)	2006–2007	Germany, Berlin–Munich	not given*	Standardized allergy patients (GA²LEN)	14.4	SPT
(Eben et al. 2008)	2005-2007	Germany, Munich	1070	Adult patients with history allergy disease	22.9	SPT + sp IgE
(Ruëff et al. 2012)	2006-2009	Germany, Munich and 3 smaller towns, Southern Bavaria	977	Patients with documented or suspected atopic disease or food allergy. Median age 43y	19.5	SPT / sp IgE
(McIntyre et al. 2010; McIntyre et al. 2012; McIntyre et al. 2011a; McIntyre et al. 2011b)	2008-2010	Germany, Munich, Bavaria	1022	Patients from Bavarian University allergy unit (with suspected sensitization to seasonal aeroallergens)	28.3	SPT + sp IGE
(Boehme et al. 2013)	2007	Germany, south-west	1039	18-66 years old	10.8	SPT and sp IgE
(Heinzerling et al. 2009)	2006–2007	Greece, Athens	not given*	Allergy patients (GA²LEN), crude rates given (not standardized)	11.7	SPT
(Nekam et al. 2011)	pre 2011	Hungary, Kecskemet (south Hungary)	50	Adult patients with respiratory allergy for at least 2 pollen seasons including symptoms during the ragweed season.	56.0	sp IgE
(Heinzerling et al. 2009)	2006–2007	Hungary, Budapest	not given*	Standardized allergy patients (GA²LEN)	53.8	SPT
(Nekam et al. 2011)	pre 2011	Hungary, Eger (north Hungary)	50	Adult patients with respiratory allergy for at least 2 pollen seasons	52.0	sp IgE
(Nekam et al. 2011)	pre 2011	Hungary, Nyiregyhaz (south Hungary)	50	Adult patients with respiratory allergy for at least 2 pollen seasons including symptoms	54.0	sp IgE

Citation	Year(s) of data collection	Location of data collection: country, place name	Study size	Population characteristics (including age, allergy/atopic patients or general population)	Sensitization rate (%)	Reactivity marker[‡]
				during the ragweed season.		
(Dervaderics et al. 2002)	pre 2002	Hungary, Paks	185	Hungarian nationals with self-reported allergy symptoms	20.0	SPT + sp IgE
(Kadocsa and Juhász 2002)	1998-1999	Hungary, Szeded (south Hungary)	394	Seasonal allergic rhinitis patients	84.8	SPT
(Asero et al. 2012)	Not given	Italy, Clinica San Carlo, north of Milan	95	Study of immigrants in Northern Italy. But data for controls from patients seen at allergy center	56.0	SPT
(Cecchi et al. 2010)	2006-2009	Italy, Florence	3086	Adult patients at allergy and clinical immunology unit	9.3	SPT
(Heinzerling et al. 2009)	2006–2007	Italy, Genoa and Palerma	not given*	Standardized allergy patients (GA^2LEN)	3.5	SPT
(Tosi et al. 2011)	1989-2008	Italy, Legnano	18594 (total 1993-2008)	Respiratory patients (>14 y) attending an allergy clinic for the first time. Annual rates of sensitization available, we restrict to the period 1993-2008.	35.0	SPT
(Goracci and Goracci 1996)	1991-1995	Italy, Livorno (Leghorn), central Italy	325	Patients at department of allergology	6.8	sp IgE
(Albertini et al. 2012)	1992-2008	Italy, Parma	19,468	Outpatients affected by respiratory disease	4.5	SPT
(Carosso and Gallesio 2000)	1996-1997	Italy, Turin	5680	Subjects affected by rhino-conjunctivitis and/or bronchial asthma during spring and summer by respiratory symptoms	9.5	sp IgE
(Asero et al. 2012)	Not given	Italy, Verona	75	Study of immigrants in Northern Italy. But data for controls from patients seen at allergy center	0.0	SPT
(Milkovska et al. 2008)	1998-2000	Macedonia, Skopje	722	Random subjects aged 18-78 (mean age 39.6y)	6.5	SPT
(Heinzerling	2006–2007	Netherlands, The; Amsterdam	not	Standardized allergy patients	18.6	SPT

Citation	Year(s) of data collection	Location of data collection: country, place name	Study size	Population characteristics (including age, allergy/atopic patients or general population)	Sensitization rate (%)	Reactivity marker[‡]
et al. 2009)			given*	(GA^2LEN)		
(Beijer and Vertegaal 2010)	2007	Netherlands, The; authors are based in Leiden and Leiderdorp	307	Participants tested positive for specific IgE for common inhalant allergies.	9.4	sp IgE
(De Weger et al. 2009)	2004-2006	Netherlands, The; Leiden?	250	Patients with suspected allergic disease. Allergic to inhaled aeroallergens	6.7	sp IgE
(Heinzerling et al. 2009)	2006–2007	Poland, Lodz	not given*	Standardized allergy patients (GA^2LEN)	10.8	SPT
(Heinzerling et al. 2009) [a]	2006–2007	Portugal, Coimbra	not given*	Standardized allergy patients (GA^2LEN)	12.4	SPT
(Popescu and Tudose 2011)	2009-2010	Romania, Bucharest	447	Patients with allergic rhinitis (5-62y)	11.2	SPT / sp IgE
(Ianovici et al. 2013)	2009	Romania, Timișoara city	1036	Patients who presented during 2009 to allergy specialist, 228 sensitized to ragweed	22.0	SPT
(Zvezdin et al. 2004)	2000, 2002, 2003	Serbia, Novi Sad	2148 (691, 756, 701)	People who had symptoms of allergic rhinitis and / or asthma	30.0	SPT
(Ackermann-Liebrich et al. 2009)	2002	Switzerland, Aarau	949	Population cohort of adults (SAPALDIA cohort)	8.3	sp IgE
(Ackermann-Liebrich et al. 2009)	2002	Switzerland, Basel	778	Population cohort of adults (SAPALDIA cohort)	10.3	sp IgE
(Ackermann-Liebrich et al. 2009)	2002	Switzerland, Geneva	398	Population cohort of adults (SAPALDIA cohort)	8.3	sp IgE
(Ackermann-Liebrich et al. 2009)	2002	Switzerland, Lugano	741	Population cohort of adults (SAPALDIA cohort)	6.9	sp IgE
(Ackermann-Liebrich et	2002	Switzerland, Payerne	810	Population cohort of adults (SAPALDIA cohort)	5.8	sp IgE

Citation	Year(s) of data collection	Location of data collection: country, place name	Study size	Population characteristics (including age, allergy/atopic patients or general population)	Sensitization rate (%)	Reactivity marker[‡]
al. 2009)						
(Ackermann-Liebrich et al. 2009)	2002	Switzerland, Wald	1095	Population cohort of adults (SAPALDIA cohort)	7.8	sp IgE
(Heinzerling et al. 2009)	2006–2007	Switzerland, Zurich	not given*	Standardized allergy patients (GA^2LEN)	18.6	SPT
(Heinzerling et al. 2009)	2006–2007	United Kingdom, London	not given*	Allergy patients (GA^2LEN), crude rates given (not standardized)	7.9	SPT

[‡] SPT: Skin prick tests; sp IgE: Ragweed specific Immunoglobulin E

* Although no specific population for each location is provided, these data are all from the GA^2LEN study which collectively included over 3000 people. Hence we assumed that each individual location would have had a large enough population to be included in our study

[a] Excluded from the statistical analysis due to a very low baseline pollen level (< 10 grains m^3y^{-1})

References

Ackermann-Liebrich U, Schindler C, Frei P, Probst-Hensch NM, Imboden M, Gemperli A, et al. 2009. Sensitisation to *Ambrosia* in Switzerland: A public health threat in waiting. Swiss Med Wkly 139:70-75.

Albertini R, Ugolotti M, Peveri S, Valenti MT, Usberti I, Ridolo E, et al. 2012. Evolution of ragweed pollen concentrations, sensitization and related allergic clinical symptoms in Parma (northern Italy). Aerobiologia 28:347-354.

Asero R, Conte M, Senna GE. 2012. Features of sensitization to airborne allergens among extra-european immigrants living in 2 distinct areas of Northern Italy. Eur Ann Allergy Clinical Immunol 44:107-112.

ATOPICA. 2014. Atopica Final Report. Brussels:European Union.

Beijer C, Vertegaal M. 2010. Sensitisation to *Ambrosia* in The Netherlands: a public health threat or just cross-reaction? Allergy 65:94-94.

Boehme MWJ, Kompauer I, Weidner U, Piechotowski I, Gabrio T, Behrendt H. 2013. Respiratory symptoms and sensitization to airborne pollen of ragweed and mugwort of adults in Southwest Germany. Dtsch Med Wochenschr 138:1651-1658.

Burbach GJ, Heinzerling LM, Edenharter G, Bachert C, Bindslev-Jensen C, Bonini S, et al. 2009. GA^{2}LEN skin test study II: Clinical relevance of inhalant allergen sensitizations in Europe. Allergy 64:1507-1515.

Carosso A, Gallesio MT. 2000. Allergy to ragweed: Clinical relevance in Turin. Aerobiologia 16:155-158.

Cecchi L, D'Amato G, Ayres JG, Galan C, Forastiere F, Forsberg B, et al. 2010. Projections of the effects of climate change on allergic asthma: The contribution of aerobiology. Allergy 65:1073-1081.

Cvitanovic S, Znaor L, Kanceljak-Macan B. 2007. Allergic rhinitis and asthma in southern Croatia:

impact of sensitization to *Ambrosia elatior*. . Croatian Medical Journal 48:68-75.

De Weger LA, Van Der Linden AC, Terreehorst I, Van Der Slikke WJ, Van Vliet AJH, Hiemstra PS. 2009. *Ambrosia* in the Netherlands: Allergic sensitivities and spreading of plants and clumps. Ned Tijdschr Geneeskd 153:798-803.

Deloraine A, Rouvière F. 2000. Place de l'allergie due à l'ambroisie parmi les pollinoses en Rhône-Alpes. Grenoble, France:Centre Rhône-Alpes d'épidémiologie et de prévention sanitaire (CAREPS).

Dervaderics M, Fust G, Otos M, Barok J, Pataky G. 2002. Differences in the sensitisation to ragweed pollen and occurrence of late summer allergic symptoms between native and immigrant workers of the Nuclear Power Plant of Hungary. Immunol Invest 31:29-40.

Eben R, Gabrio T, Huss-Marp J, Darsow U, Jessberger B, Link B, et al. 2008. Sensitisation to *Ambrosia artemisiifolia* (ragweed): a common finding in Southern Germany with potential clinical relevance. Allergy 63:466-467.

Gabrio T, Alberternst B, Böhme M, Kaminski U, Nawrath S, Behrendt H. 2010. Sensitization to allergens of *Ambrosia artemisiifolia* and other allergens on 10 years old children and adults in Baden-Württemberg. Umweltmed Forsch Prax 15:15-22.

Goracci E, Goracci G. 1996. Ragweed (*Ambrosia*) pollen presence in Livorno, Central Italy: Aerobiological and sensitization data. Aerobiologia 12:139-170.

Heinzerling LM, Burbach GJ, Edenharter G, Bachert C, Bindslev-Jensen C, Bonini S, et al. 2009. GA^{2}LEN skin test study I: GALEN harmonization of skin prick testing: Novel sensitization patterns for inhalant allergens in Europe. Allergy 64:1498-1506.

Hemmer W, Schauer U, Trinca A, Neumann C, Jarisch R. 2011. Ragweed Pollen Allergy in Austria: a Retrospective Analysis of Sensitization Rates from 1997 to 2007. J Allergy Clin Immunol 127:AB170-AB170.

Ianovici N, Panaitescu CB, Brudiu I. 2013. Analysis of airborne allergenic pollen spectrum for 2009 in Timişoara, Romania. Aerobiologia 29:95-111.

Jäger S. 2000. Ragweed (*Ambrosia*) sensitisation rates correlate with the amount of inhaled airborne pollen. A 14-year study in Vienna, Austria. Aerobiologia 16:149-153.

Kadocsa E, Juhász M. 2002. Study of airborne pollen composition and allergen spectrum of hay fever patients in South Hungary (1990-1999). Aerobiologia 18:203-209.

McIntyre M, Jaeger T, Wopfner N, Ferreira F, Ollert M, Ring J, et al. 2010. Ragweed testing in Bavaria: positive challenge test result in every fourth patient with *Ambrosia* sensitisation. Allergy 65:200-201.

McIntyre M, Jaeger T, Huss-Marp J, Hauser M, Ferreira F, Ollert M, et al. 2012. 30 Ragweed Allergy – What Role Does It Play in Bavaria? The World Allergy Organization Journal 5:S27-S28.

McIntyre MS, Jaeger T, Huss-Marp J, Ollert M, Schmidt-Weber C, Ring J, et al. 2011a. Ragweed sensitization in Bavaria: Results of a three-year Study on clinical Relevance. Journal Der Deutschen Dermatologischen Gesellschaft 9:132-132.

McIntyre MS, Jaeger T, Huss-Marp J, Wopfner N, Ferreira F, Ollert M, et al. 2011b. Sensitisation and allergy to ragweed in Bavaria - a health threat? Allergy 66:250-250.

Mehulić M, Mehulić K, Vuljanko IM, Kukulj S, Grle SP, Vukić AD, et al. 2011. Changing pattern of sensitization in Croatia to aeroallergens in adult population referring to allergy clinic during a period of 15 years. Coll Antropol 35:529-536.

Milkovska S, Karadzinska-Bislimovska J, Cvetanov V, Janackov B, Minov J, Risteska-Kuc S, et al. 2008. *Ambrosia* and *Artemisia* in r. Macedonia: aeropallinological and allergenic significance. Allergy 63:398-398.

Nekam K, Paldy A, Apatini D, Magyar D, DuBuske LM. 2011. Sensitization Patterns to Allergens in Ragweed Allergic Patients from Regions of Hungary Having Different Levels of Ragweed Allergen Exposure. J Allergy Clin Immunol 127:AB255-AB255.

Nikolov G, Hristova-Savova M, Nedyalkov M, Petrunov B. 2013. The Role of Ambrosia Allergen in respiratory diseases in Bulgaria. Allergy, Asthma & Immunophysiology: From Basic Science to Clinical Management:35-38.

Peternel R, Milanović SM, Srnec L. 2008. Airborne ragweed (*Ambrosia artemisiifolia L.*) pollen content in the city of Zagreb and implications on pollen allergy. Ann Agric Environ Med 15:125-130.

Popescu F, Tudose AM. 2011. Ambrosia pollen sensitization in allergic rhinitis patients from the central part of the Romanian Plain Romanian Journal of the Rhinology 1:26-30.

Ruëff F, Przybilla B, Walker A, Gmeiner J, Kramer M, Sabanés-Bové D, et al. 2012. Sensitization to common ragweed in southern bavaria: Clinical and geographical risk factors in atopic patients. Int Arch Allergy Immunol 159:65-74.

Rybníček O, Novotná B, Rybníčkova E, Rybníček K. 2000. Ragweed in the Czech Republic. Aerobiologia 16:287-290.

Tosi A, Wüthrich B, Bonini M, Pietragalla-Köhler B. 2011. Time lag between *Ambrosia* sensitisation and *Ambrosia* allergy: A 20-year study (1989-2008) in Legnano, northern Italy. Swiss Med Wkly 141.

Toth I, Peternel R, Gajnik D, Vojniković B. 2011. Micro-regional hypersensitivity variations to inhalant allergens in the city of Zagreb and Zagreb county. Coll Antropol 35:31-37.

Zvezdin B, Radišić P, Kojičić M, Obradović-Anđelić S, Jarić D, Tepavac A, et al. 2004. Alergijske bolesti respiratornog trakta i polen ambrozije kao njihov uzročni faktor [AMBROSIA POLLEN - A CAUSATIVE AGENT OF ALLERGIC DISEASES OF THE RESPIRATORY TRACT]. Pneumon 4.

Figure S2; Baseline and future population (percentage) sensitized to ragweed pollen at the NUTS2 level for RCP4.5 and RCP8.5 and a reference plant invasion scenario. Data are presented separately for the CHIMERE and WRF/RegCM model suites. Data source: GISCO - Eurostat (European Commission). Administrative boundaries: © EuroGeographics © UN-FAO © Turkstat.

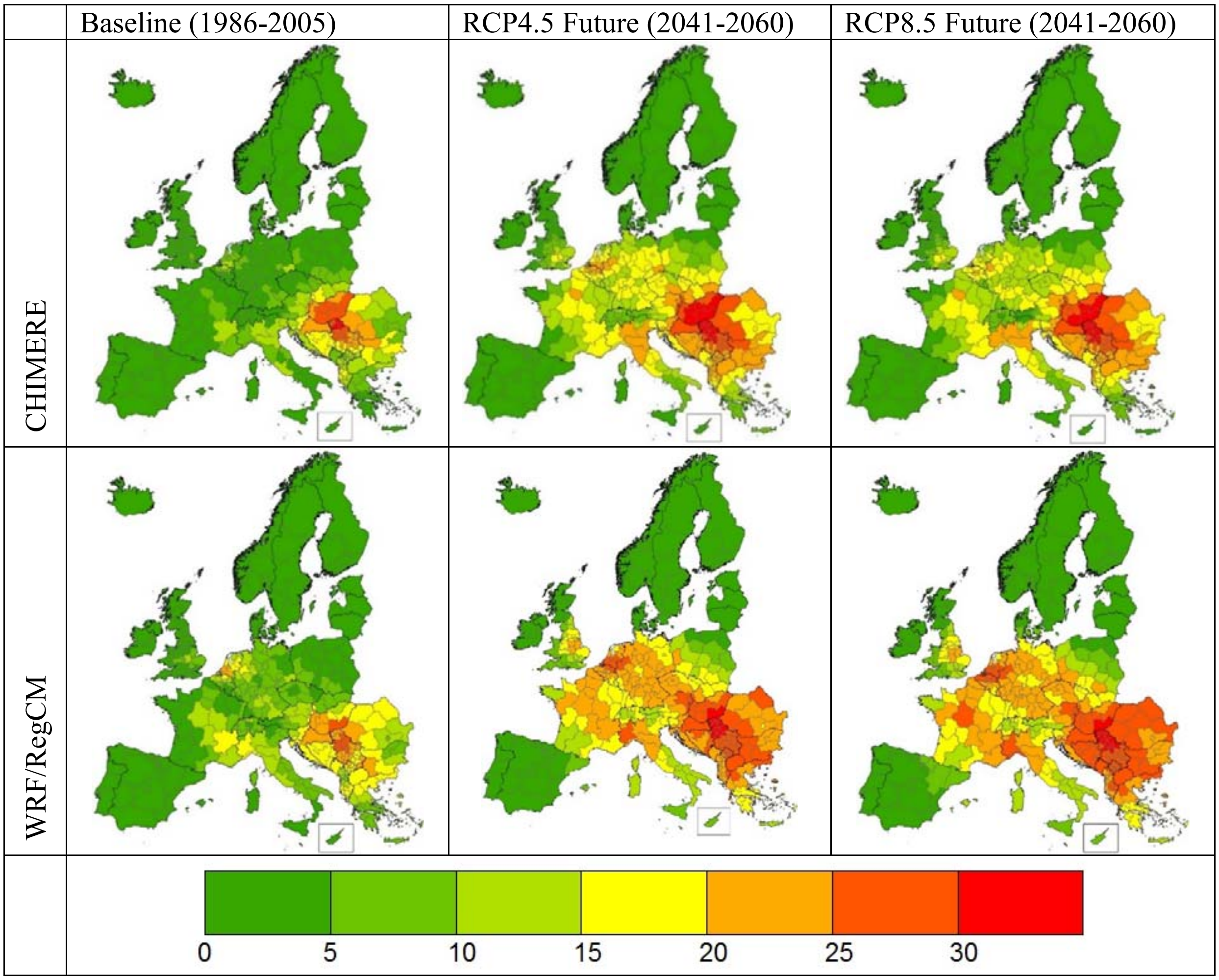

Table S3: Population (in thousands) sensitized to ragweed for the current and future. Reference ragweed invasion scenario comparing RCP 4.5 and RCP 8.5, and 2 climate model suites (Chimere, WRF/RegCM) for 40 European countries; 95% confidence interval (CIs) shown in brackets.

Country	Population sensitized in thousands					
	Chimere			WRF/RegCM		
	Baseline	2041-2060		Baseline	2041-2060	
		RCP4.5	RCP8.5		RCP4.5	RCP8.5
Austria	868 (58,1883)	1636 (342,3017)	1544 (307,2891)	912 (42,2020)	1863 (419,3328)	1814 (392,3262)
Belgium	732 (0,1934)	2143 (358,3939)	1997 (275,3738)	1115 (44,2525)	2585 (623,4547)	2693 (691,4696)
Bulgaria	1135 (110,2254)	1614 (330,2912)	1692 (368,3020)	1165 (146,2296)	1912 (502,3322)	2025 (573,3478)
Croatia	804 (139,1513)	1041 (243,1839)	1018 (229,1807)	943 (187,1704)	1156 (315,1997)	1157 (316,1999)
Cyprus	0 (0,2)	1 (0,59)	0 (0,50)	15 (0,91)	71 (0,172)	82 (0,187)
Czech Republic	409 (0,1484)	1756 (152,3377)	1615 (90,3183)	565 (2,1739)	2130 (371,3891)	2145 (378,3912)
Denmark	0 (0,0)	29 (0,412)	8 (0,306)	0 (0,58)	163 (0,729)	229 (0,822)
Estonia	0 (0,0)	0 (0,1)	0 (0,8)	0 (0,0)	0 (0,2)	0 (0,6)
Finland	0 (0,0)	0 (0,0)	0 (0,0)	0 (0,0)	0 (0,0)	0 (0,0)
France	2256 (41,6228)	8849 (981,17862)	8210 (866,16840)	4210 (145,11127)	12582 (2306,23050)	13233 (2650,23945)
Germany	2282 (0,8849)	13337 (1267,25830)	11814 (726,23735)	7095 (114,17219)	18041 (3847,32297)	18033 (3831,32287)
Greece	487 (5,1687)	1341 (69,2878)	1179 (50,2656)	1176 (50,2652)	2188 (336,4043)	2229 (360,4099)
Hungary	2668 (750,4586)	3069 (1000,5137)	3057 (993,5121)	1910 (328,3544)	2729 (788,4670)	2744 (798,4690)
Ireland	0 (0,0)	0 (0,0)	0 (0,0)	0 (0,0)	8 (0,263)	1 (0,144)
Italy	4097 (113,10583)	9563 (1409,18679)	9174 (1449,18144)	5474 (300,13055)	10656 (1545,20182)	10923 (1649,20548)
Latvia	0 (0,0)	0 (0,82)	0 (0,114)	0 (0,0)	0 (0,64)	0 (0,106)
Lithuania	0 (0,20)	11 (0,273)	29 (0,320)	0 (0,0)	2 (0,225)	4 (0,267)
Luxembourg	0 (0,4)	57 (0,125)	48 (0,112)	31 (0,88)	98 (15,181)	102 (18,187)
Malta	0 (0,13)	18 (0,63)	10 (0,52)	0 (0,37)	38 (0,90)	37 (0,88)
Netherlands	1300 (0,3224)	2863 (346,5455)	2494 (197,4947)	3148 (487,5847)	4115 (1054,7177)	4256 (1142,7371)
Poland	1251 (0,4590)	4175 (221,9237)	4087 (217,9115)	994 (4,4182)	4619 (343,9848)	4889 (443,10218)
Portugal	0 (0,0)	0 (0,0)	0 (0,0)	0 (0,0)	0 (0,0)	0 (0,0)
Romania	3045 (392,6154)	4473 (864,8117)	4802 (1044,8570)	3148 (332,6296)	5072 (1203,8941)	5551 (1502,9600)
Slovakia	790 (65,1583)	1221 (266,2175)	1210 (259,2160)	462 (16,1133)	1100 (191,2009)	1177 (239,2116)
Slovenia	281 (17,574)	397 (61,734)	393 (59,728)	327 (30,638)	451 (95,808)	449 (93,805)
Spain	0 (0,2)	35 (0,843)	10 (0,632)	42 (0,745)	858 (0,3278)	1372 (0,4358)
Sweden	0 (0,0)	1 (0,121)	0 (0,94)	0 (0,3)	25 (0,213)	45 (0,283)
United Kingdom	1008 (0,3774)	5113 (244,11626)	4568 (223,10689)	1384 (33,6270)	7232 (488,15281)	7378 (530,15402)
**Sum EU28**	**23413 (1691,60938)**	**62743 (8153,124793)**	**58959 (7351,119034)**	**34116 (2263,83266)**	**79693 (14442,150607)**	**82569 (15603,154876)**
Albania	400 (9,812)	580 (101,1059)	559 (88,1031)	388 (10,795)	673 (159,1186)	703 (178,1228)
Andorra	0 (0,0)	0 (0,5)	0 (0,4)	0 (0,4)	5 (0,14)	7 (0,17)
Bosnia & Herzegovina	682 (88,1289)	869 (193,1545)	848 (179,1516)	724 (104,1346)	958 (248,1668)	977 (260,1694)
Iceland	0 (0,0)	0 (0,0)	0 (0,0)	0 (0,0)	0 (0,0)	0 (0,0)
Kosovo	196 (1,429)	391 (85,697)	382 (79,684)	323 (43,603)	468 (133,802)	471 (135,808)
Liechtenstein	0 (0,0)	1 (0,4)	1 (0,4)	1 (0,5)	4 (0,9)	4 (0,9)
FYR Macedonia	291 (5,588)	449 (92,805)	427 (79,776)	308 (19,612)	524 (139,908)	525 (140,910)
Montenegro	56 (0,133)	101 (7,195)	103 (8,199)	101 (9,196)	146 (35,258)	155 (40,270)
Norway	0 (0,0)	0 (0,0)	0 (0,0)	0 (0,0)	0 (0,13)	0 (0,17)
San Marino	2 (0,6)	5 (0,9)	4 (0,9)	2 (0,6)	4 (0,9)	5 (0,9)
Serbia	1731 (427,3045)	2042 (611,3472)	2035 (607,3463)	1685 (388,2981)	2105 (651,3559)	2121 (661,3581)
Switzerland	135 (0,654)	650 (3,1607)	598 (4,1535)	618 (1,1563)	1257 (117,2442)	1263 (116,2449)
**Sum non-EU28**	**3493 (530,6955)**	**5087 (1092,9400)**	**4957 (1044,9221)**	**4151 (575,8111)**	**6144 (1482,10869)**	**6231 (1530,10992)**
**Sum Europe**	**26905 (2222,67893)**	**67829 (9245,134193)**	**63916 (8395,128255)**	**38266 (2838,91377)**	**85837 (15923,161476)**	**88799 (17133,165868)**

Table S4: Current and future populations clinically sensitized to ragweed pollen. Changes due to population change are also presented. Data are average of the CHIMERE and WRF/RegCM model suites for RCP4.5 and a reference plant invasion scenario.

Country	Clinical sensitization value (%)[a]	Projected change in population, baseline to 2050 (%)	Population clinically sensitized in thousands [CHIMERE, WRF/RegCM][*]					
			Baseline		2041-2060		2041-2060 plus population change	
Austria	62.4	7.8	555	[542,569]	1092	[1021,1163]	1176	[1101,1254]
Belgium	100	9.4	923	[732,1115]	2364	[2143,2585]	2585	[2344,2828]
Bulgaria	75.9[b]	-31.2	873	[861,884]	1338	[1225,1451]	920	[843,998]
Croatia	75.9[b]	-19.9	663	[610,716]	834	[790,877]	668	[633,702]
Cyprus	75.9[b]	22.9	6	[0,11]	27	[1,54]	33	[1,66]
Czech Republic	75.9[b]	5.4	370	[310,429]	1475	[1333,1617]	1554	[1405,1704]
Denmark	64.6	12.5	0	[0,0]	62	[19,105]	70	[21,118]
Estonia	75.9[b]	-14.2	0	[0,0]	0	[0,0]	0	[0,0]
Finland	60.9	5.3	0	[0,0]	0	[0,0]	0	[0,0]
France	50.0	12.6	1616	[1128,2105]	5358	[4425,6291]	6035	[4983,7084]
Germany	83.6	-15.9	3919	[1908,5931]	13116	[11150,15082]	11037	[9377,12684]
Greece	43.6	-7.0	363	[212,585]	769	[513,954]	715	[477,887]
Hungary	92.4	-11.5	2115	[2465,1765]	2678	[2836,2522]	2370	[2510,2232]
Ireland	75.9[b]	29.2	0	[0,0]	3	[0,6]	4	[0,8]
Italy	88.6	-2.4	4240	[3630,4850]	8957	[8473,9441]	8738	[8270,9214]
Latvia	75.9[b]	-22.5	0	[0,0]	0	[0,0]	0	[0,0]
Lithuania	75.9[b]	-19.8	0	[0,0]	5	[8,2]	4	[6,2]
Luxembourg	75.9[b]	37.1	12	[0,24]	59	[43,74]	81	[59,101]
Malta	75.9[b]	-2.1	0	[0,0]	21	[14,29]	21	[14,28]
Netherlands	89.8	-0.6	1997	[1167,2827]	3133	[2571,3695]	3115	[2556,3673]
Poland	50.0	-11.3	561	[626,497]	2199	[2088,2310]	1951	[1852,2049]
Portugal	87.1	-8.8	0	[0,0]	0	[0,0]	0	[0,0]
Romania	75.9[b]	-19.6	2350	[2311,2389]	3622	[3395,3850]	2913	[2730,3095]
Slovakia	75.9[b]	-8.4	475	[600,351]	881	[927,835]	807	[849,765]
Slovenia	75.9[b]	-3.4	231	[213,248]	322	[301,342]	311	[291,330]
Spain	75.9[b]	1.6	16	[0,32]	339	[27,651]	345	[27,661]
Sweden	75.9[b]	25.8	0	[0,0]	10	[1,19]	12	[1,24]
UK	89.9	16.4	1075	[906,1244]	5549	[4597,6502]	6462	[5351,7568]
**Sum EU28**			**22361**	**[18221,26572]**	**54212**	**[47901,60457]**	**51928**	**[47901,60457]**
Albania	75.9[b]	-6.0	299	[304,294]	475	[440,511]	447	[414,480]
Andorra	75.9[b]	21.9	0	[0,0]	2	[0,4]	2	[0,5]
Bosnia & Herzegovina	75.9[b]	-13.4	534	[518,550]	693	[660,727]	601	[572,630]
Iceland	75.9[b]	27.0	0	[0,0]	0	[0,0]	0	[0,0]
Kosovo	75.9[b]	29.5	197	[149,245]	326	[297,355]	422	[385,460]
Liechtenstein	75.9[b]	21.8	0	[0,1]	2	[1,3]	2	[1,4]
FYR Macedonia	75.9[b]	-10.5	227	[221,234]	369	[341,398]	330	[305,356]
Montenegro	75.9[b]	-10.2	60	[43,77]	94	[77,111]	84	[69,100]
Norway	75.9[b]	33.1	0	[0,0]	0	[0,0]	0	[0,0]
San Marino	75.9[b]	6.9	2	[2,2]	3	[4,3]	4	[4,3]
Serbia	75.9[b]	-30.9	1296	[1314,1279]	1574	[1550,1598]	1087	[1071,1104]
Switzerland	52.2	34.4	196	[70,323]	498	[339,656]	669	[456,882]
**Sum non-EU28**			**2811**	**[2621,3005]**	**4036**	**[3709,4366]**	**3649**	**[3709,4366]**
**Sum Europe**			**25172**	**[20842,29577]**	**58248**	**[51610,64823]**	**55577**	**[51610,64823]**

[a] Country level estimates of the proportion of ragweed sensitized individuals who will experience clinical symptoms. Data from Burbach et al. (2009). Where country specific data is not available [b] the European average value of 75.9% is used
